# A Multi-Disciplinary Approach to Managing Anterior Aesthetics: Crown Lengthening/Digital Smile Design (DSD)/Computer-Aided Design and Computer-Aided Manufacturing (CAD/CAM)

**DOI:** 10.7759/cureus.85961

**Published:** 2025-06-13

**Authors:** Siham Kouji, Amal El Yamani

**Affiliations:** 1 Department of Fixed Prosthodontics, Faculty of Dental Medicine of Rabat, Mohammed V University, Rabat, MAR

**Keywords:** case report, computer-aided design/computer-aided manufacturing technology (cad-cam), crown lengthening, dental smile design dsd, esthetics management, full anterior ceramic crowns

## Abstract

The restoration of anterior teeth has always been a significant challenge for both the dentist and the laboratory technician. Achieving the final result of an aesthetic prosthetic project requires a careful reflection phase upstream, involving close collaboration between the medical team, following several specific steps, and adhering to a precise chronology. This case report highlights the importance of a multidisciplinary treatment approach, including crown lengthening surgery with osteotomy, pre-visualization of the final result using Digital Smile Design software, and computer-aided design and computer-aided manufacturing (CAD/CAM) ceramic crowns for optimal correction of smile aesthetics.

## Introduction

A natural and aesthetic appearing smile is a combination of several crucial elements [1]. There must be a harmonious relationship between tooth size, shape, proportion, and the periodontium.

Nowadays, thanks to digital progress, dentists have various digital tools at their disposal which will assist them in the various stages from planning of the aesthetic projects to their realization.

Digital Smile Design (DSD) [2] is a specialized dental treatment planning tool that enables clinicians to digitally design and modify a patient's smile, providing a visual mock-up of the anticipated results before initiating any physical procedures.

Computer-aided design and computer-aided manufacturing (CAD/CAM) technology has significantly enhanced the quality of fixed dental prostheses by enabling precise design and fabrication processes. This advancement allows for the production of restorations with improved fit, durability, and aesthetic outcomes compared to traditional methods. The integration of CAD/CAM systems streamlines workflows, reduces human error, and facilitates the use of high-quality materials, leading to more reliable and patient-specific dental solutions.

This case report highlights the significance of a comprehensive treatment approach combining crown lengthening surgery with osteotomy, DSD for pre-visualization, and CAD/CAM ceramic crowns to achieve optimal smile aesthetics.

## Case presentation

A 22-year-old patient presented to the department for anterior sector aesthetic rehabilitation. He had no significant medical history and no history of smoking or alcohol use.

Extraoral examination revealed no significant findings. His face was symmetrical with a straight profile. His smile line extended to the first molars. However, examination of the gingiva revealed gingivitis with non-alignment of the necks of the anterior incisors.

The patient received a thorough clinical evaluation, including an analysis of the occlusion and masticatory system to assess the condition of the temporomandibular joints, masticatory muscles, and overall occlusal function.

Smile aesthetics were evaluated by assessing various factors, including the initial shade, alignment of dental and facial midlines, the width-to-height ratio of the anterior teeth, the curve of Spee in relation to the lower lip during a smile, the buccal corridor, the free gingival margins, and the position of the incisal edge relative to the lips at rest or during a full smile, as well as its interaction with the F/V sounds.

A thorough periodontal assessment was conducted, encompassing measurements of probing depth, clinical attachment level, bleeding on probing, plaque index, as well as evaluations of crown and bone height. The examination was supplemented with preliminary photographs and diagnostic casts.

The examination revealed that the masticatory muscles were functioning normally. Occlusal analysis indicated a Class I molar relationship; however, anterior guidance was absent due to coronal destruction of the incisors.

**Figure 1:**
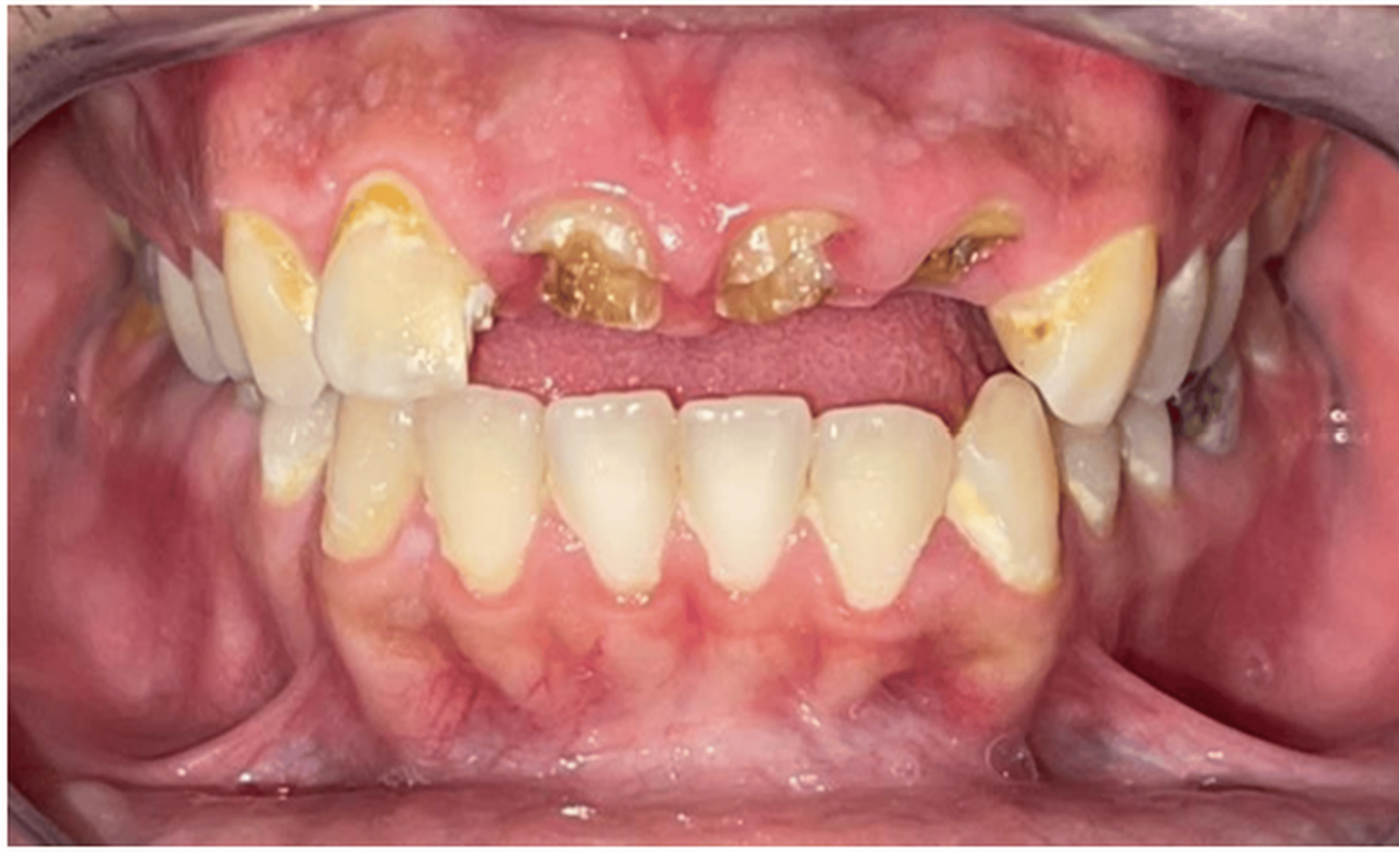
The initial situation of the clinical case. Initial dental arch exhibiting aesthetic concerns: exposed central incisors with root visibility, accompanied by caries and gingival inflammation

Lateral incisor 12 shows mesial caries attaining the pulp, with generalized demineralization and a white lesion on all maxillary and mandibular teeth. The initial shade was Vita A3 (Figure 1).

**Figure 2:**
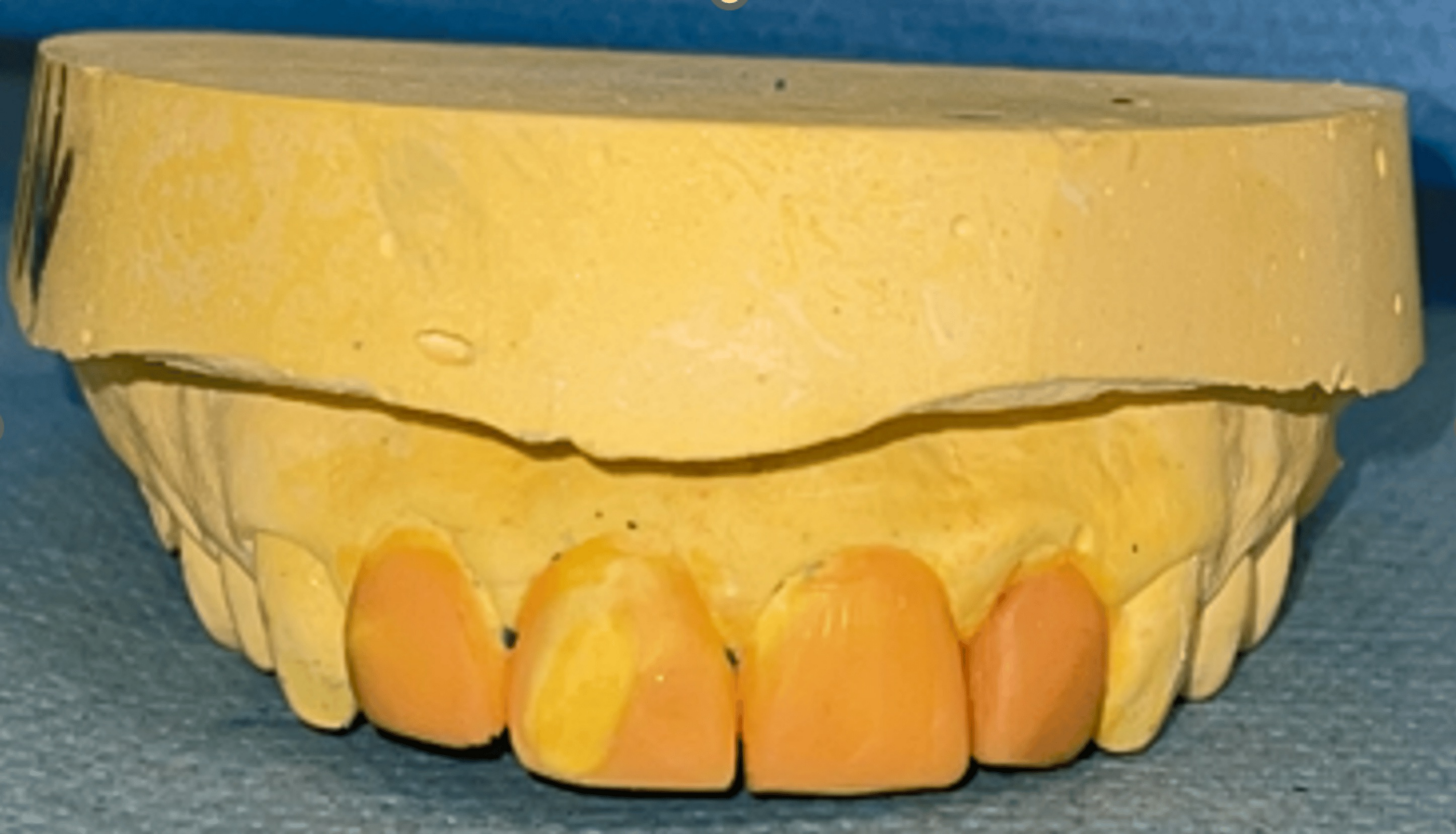
Diagnostic wax-up. Resin-based diagnostic wax-up illustrating the proposed restorative design for aesthetic and functional evaluation.

**Figure 3:**
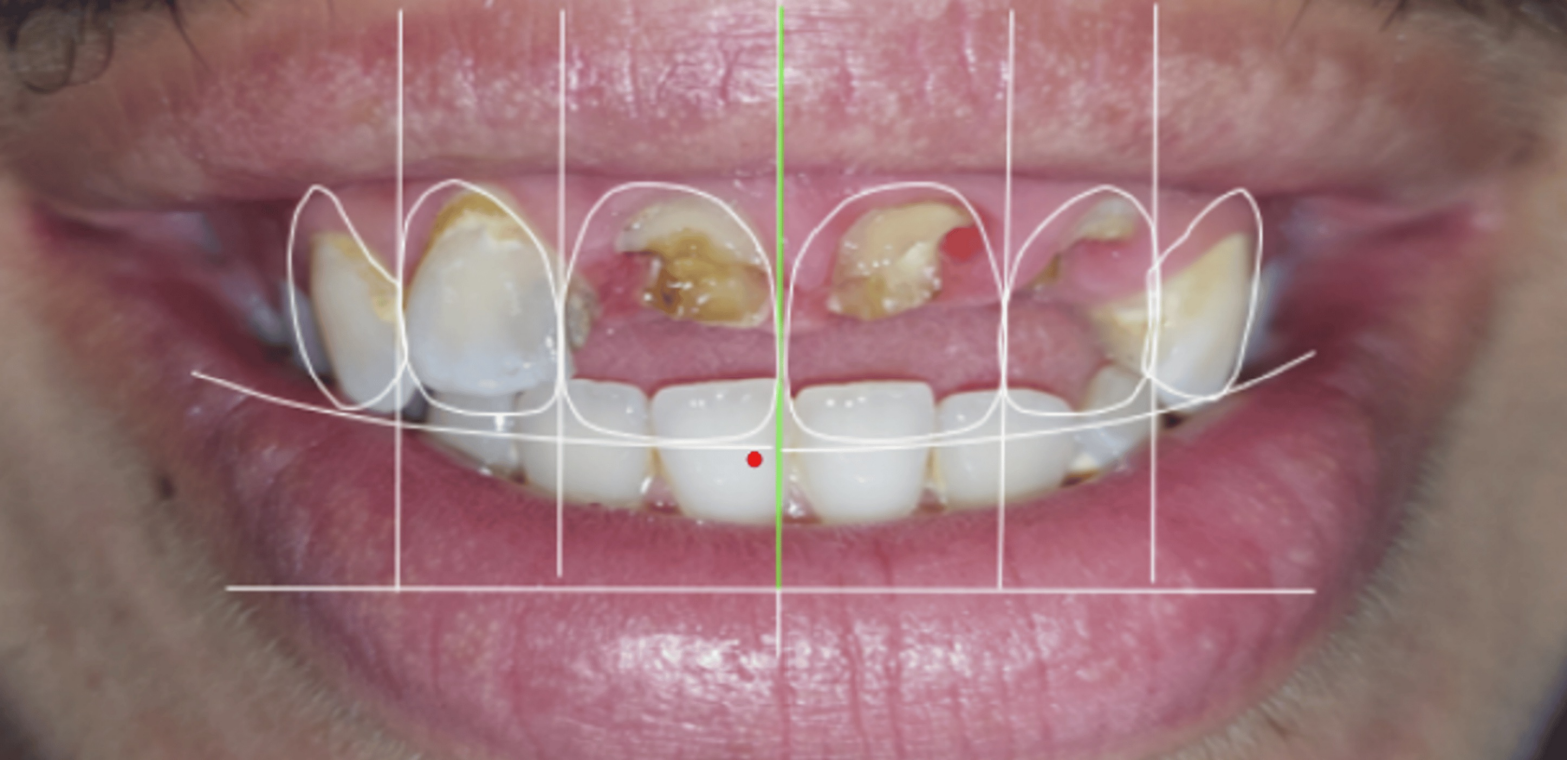
Integrated tooth and gingival contour design during the Digital Smile Design (DSD) planning phase. Dental smile design-based treatment planning emphasizing the harmony between dental contours and facial proportions.

Utilizing this data, a diagnostic wax-up (Figure 2) based on DSD (Figure 3) was created and mounted on a semi-adjustable articulator. This setup allowed for the visualization of the "ideal" tooth morphology, facilitating precise planning and communication for the esthetic rehabilitation.

After diagnosis and case analysis, the treatment plan discussed with the patient was as follows: surgical crown lengthening and neck alignment by flap surgery and bone remodelling of teeth 11,21,22 based on the diagnostic wax-up and retroalveolar cliches. Endodontic treatment of maxillary incisors with CAD/CAM ceramic crowns.

Treatment process

Surgical Technique

**Figure 4:**
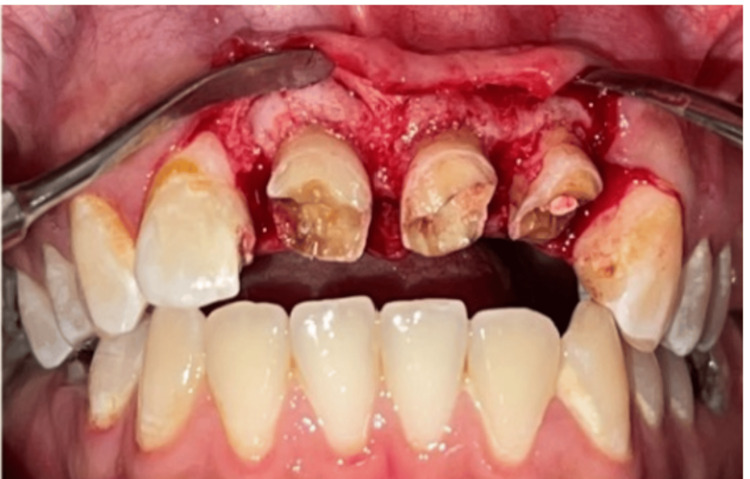
Detachment of full-thickness flaps. Reflection of a full-thickness flap to expose osseous structures during periodontal surgery.

**Figure 5:**
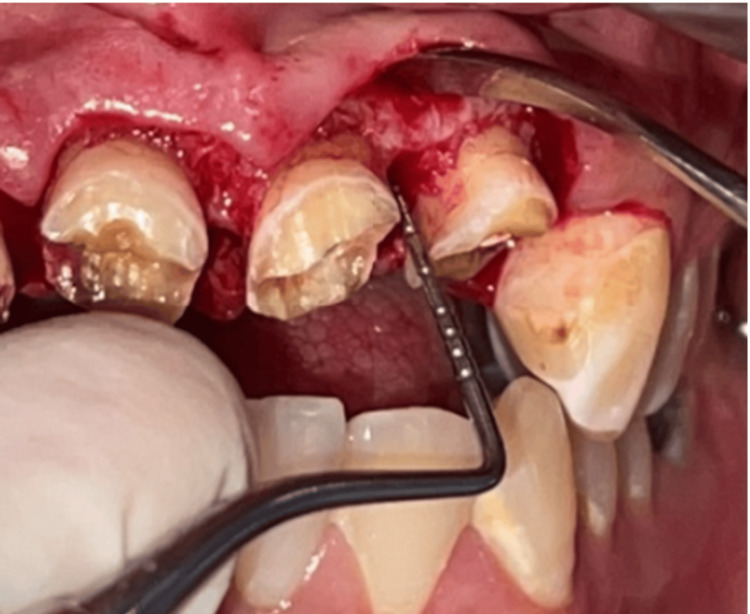
Osteotomy measurement. Measurement of the osteotomy wedge to determine the required correction in bone alignment.

**Figure 6:**
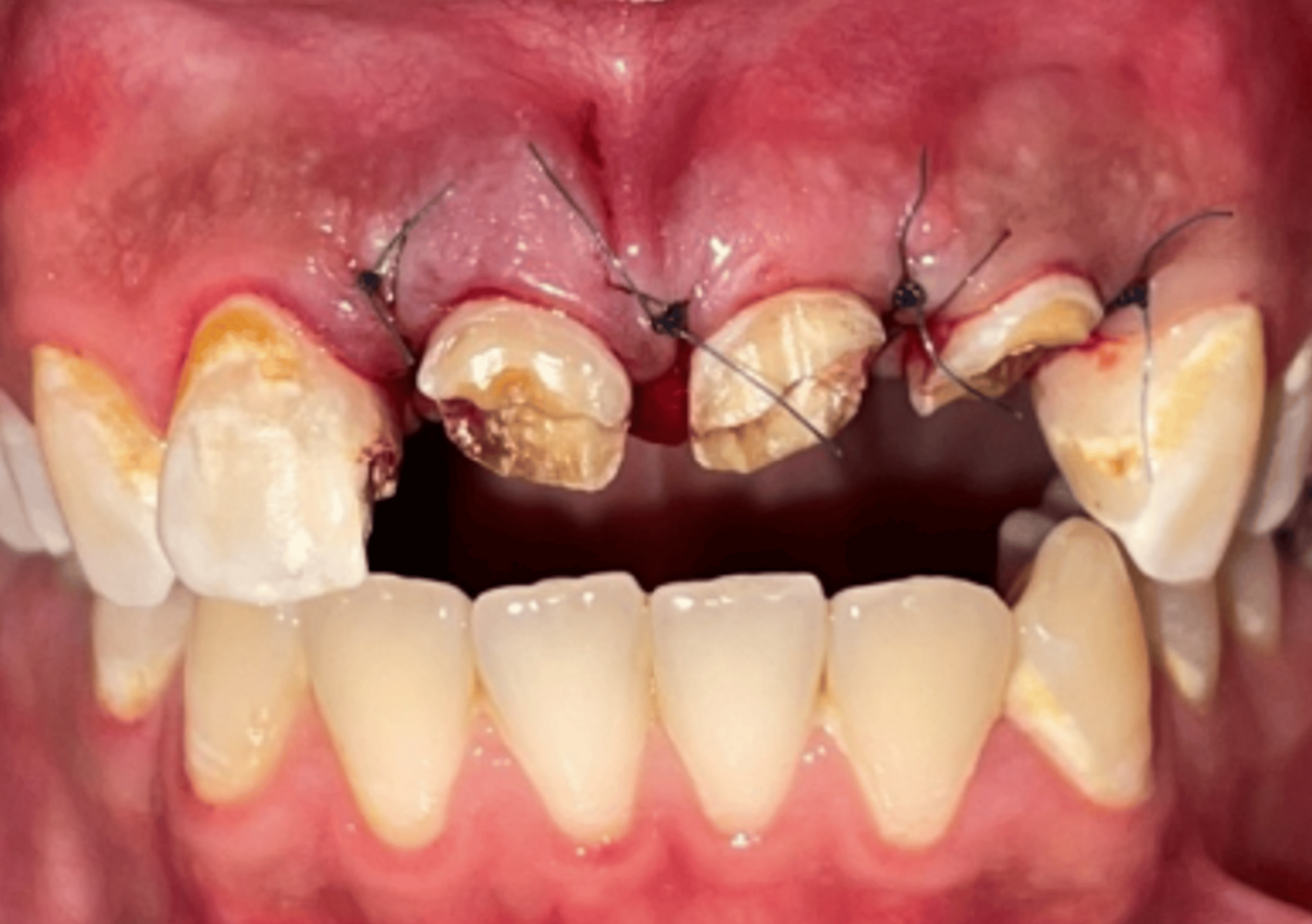
Flap is repositioned and sutured. The flap is meticulously repositioned to its original or slightly modified position and secured with sutures to facilitate optimal healing.

**Figure 7:**
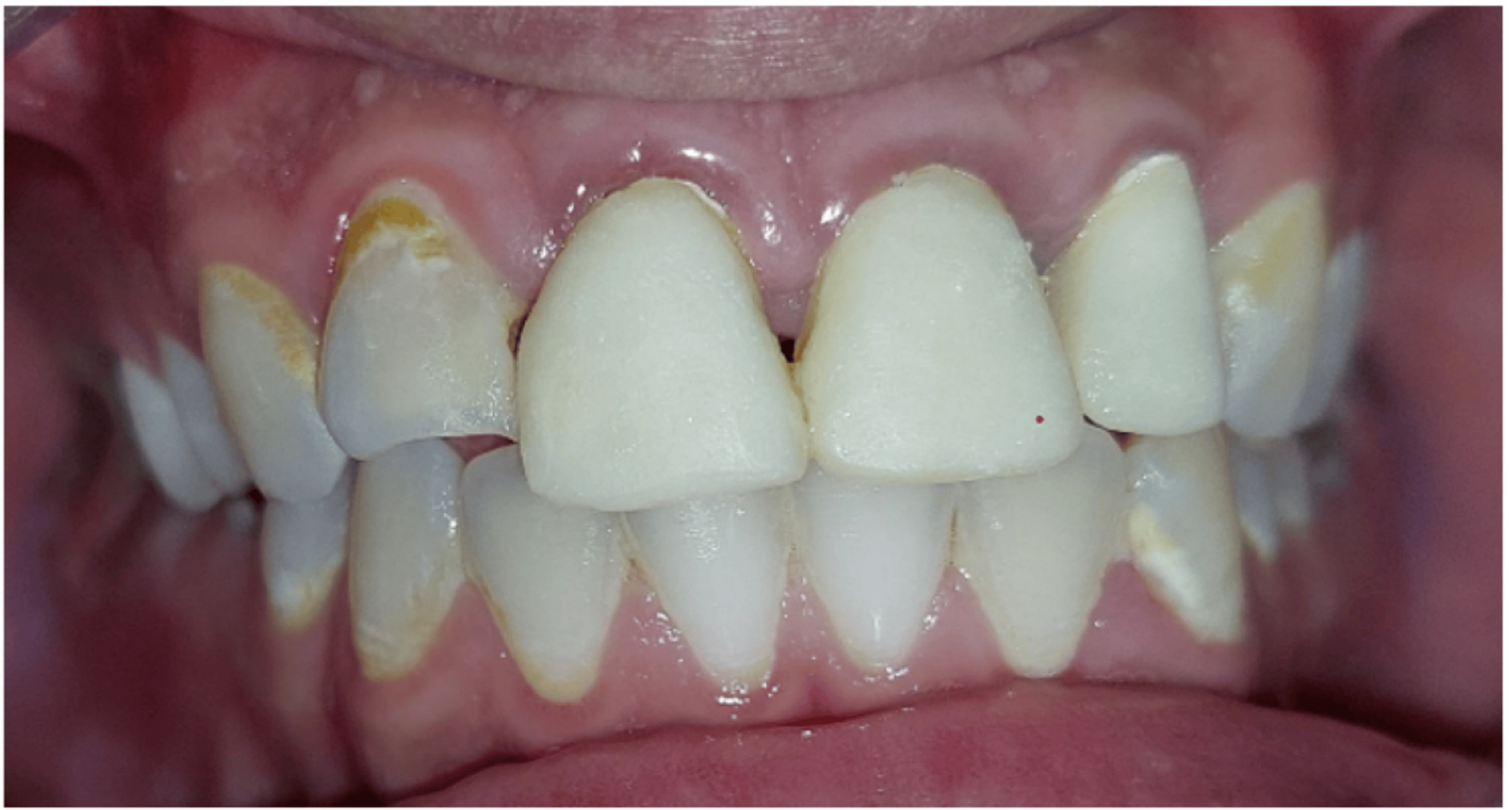
Placement of temporary prosthesis. Temporary dental prostheses: enhancing appearance and function and guiding tissue recovery

The main objective of the surgical phase is to perform a 2-mm gingivectomy on the vestibular and palatal surfaces and a 2-mm osteotomy on the mesial and distal surfaces (Figures 4, 5). Then the flap is repositioned and sutured (Figure 6). A provisional prosthesis is placed (Figure 7).

Prior to initiating surgical intervention, a hermetic endodontic treatment was performed, followed by the creation of a physical resin mock-up derived from the dental smile design analysis.

The sutures were removed one week after surgery. The patient was instructed on how to maintain strict plaque control throughout the period of cicatrization and treatment.

Teeth Preparation

Following a healing period, metal post and core restorations were indicated for the maxillary incisors due to significant coronal destruction, necessitating reinforcement to support full-coverage crowns. There is a significant loss of coronal tooth structure due to extensive decay. A post is inserted into the pre-treated root canal for enhanced retention [3]. The core is the superstructure of the root and provides retention and resistance for the artificial crown.

To prepare for the ceramic crown, a shoulder margin with a rounded internal angle was established, ensuring a smooth and continuous finish line that facilitates optimal seating and minimizes stress concentrations on both the tooth structure and the restoration, with a thickness of 1 mm on the vestibular side, 1.2 mm on the palatal side, and 1 mm on the interproximal sides.

Before the secondary impression is taken, a gingival retraction thread is placed on the prepared teeth to ensure gingival deflection and better visibility of the preparation margin.

CAD/CAM Procedures and Restoration Design

CAD [4] software is a crucial tool since it is responsible for guiding automated machines that create objects and assemblies in a virtual environment.

**Figure 8:**
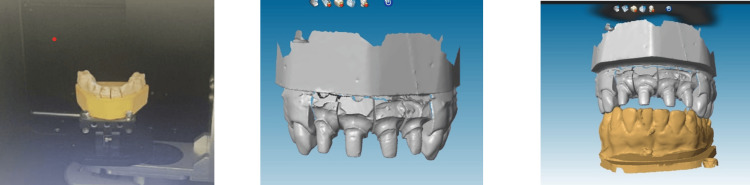
Model scanning. Scanning procedure involving maxillary and mandibular models, both individually and in occlusion.

CAD is the phase corresponding to the digital design of the prosthesis. It can be subdivided into two phases: a data treatment phase and a modeling phase (Figure 8).

During the data processing phase, the 3D model resulting from the acquisition (light scan of the plaster model) can be viewed from all angles and in all sizes. It can be reworked (trimmed, corrected, etc.), on which the future prosthesis will be based: this is the modeling phase.

**Figure 9:**
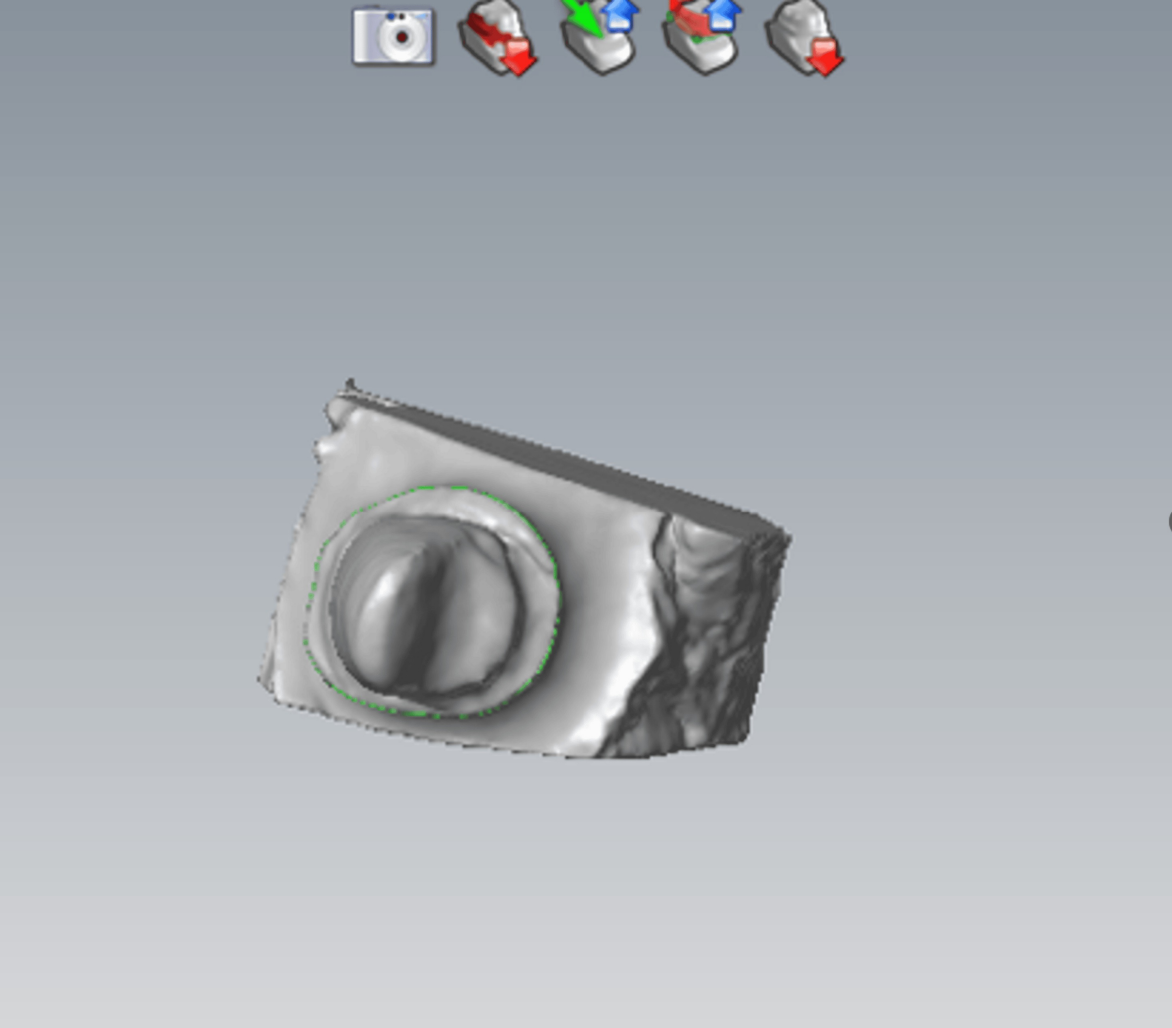
Finishing line trace: possible touch-ups. Adjusting the cervical margin within CAD software to enhance the fit and aesthetics of the dental restoration.

**Figure 10:**
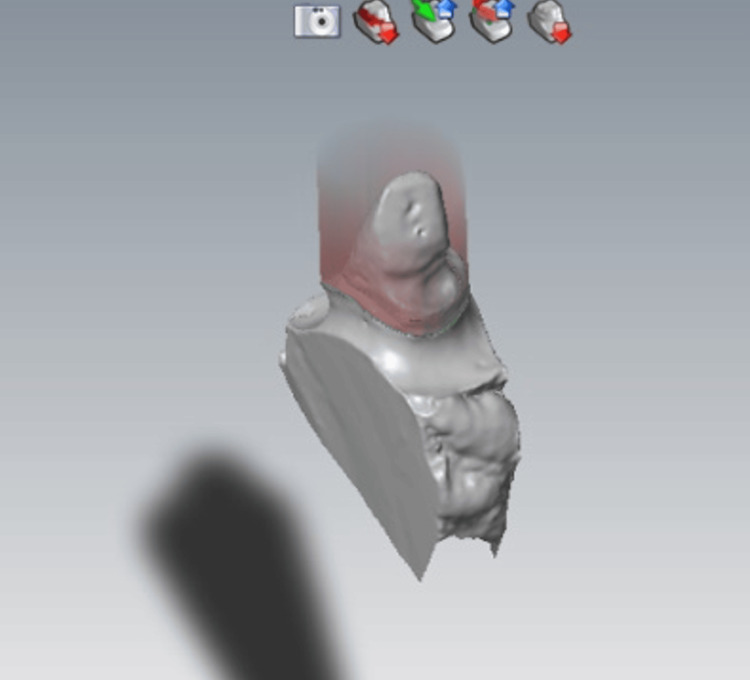
Choice of insertion axis. Adjusting the insertion axis in CAD software to ensure optimal fit and alignment of the dental restoration.

The finishing line and insertion axis are materialized, and then the CAD software proposes preforms which are tested and then adapted to the M.P.U (Figures 9, 10).

The operator can intervene on all the general characteristics of the prosthesis: on the occlusal thickness, on the cervical limits, on the spacing between the prosthesis and the working model (space left for cements or glues), etc., so the operator can completely redesign and modify the prosthesis proposed by the CAD system according to the clinical situation.

**Figure 11:**
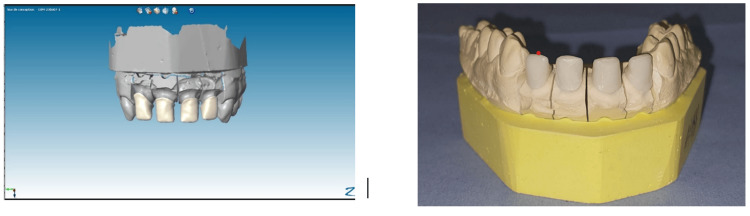
Modeling: automatic proposal (dental morphology database). Once the CAD model is finalized, CAM technology is employed to fabricate the dental prosthesis using milling machines or 3D printers.

Once the modeling is complete, the CAM software transforms the digital data into the analog motion produced by a machine tool. These machine tools allow material to be removed by cutting or milling (Figure 11).

**Figure 12:**
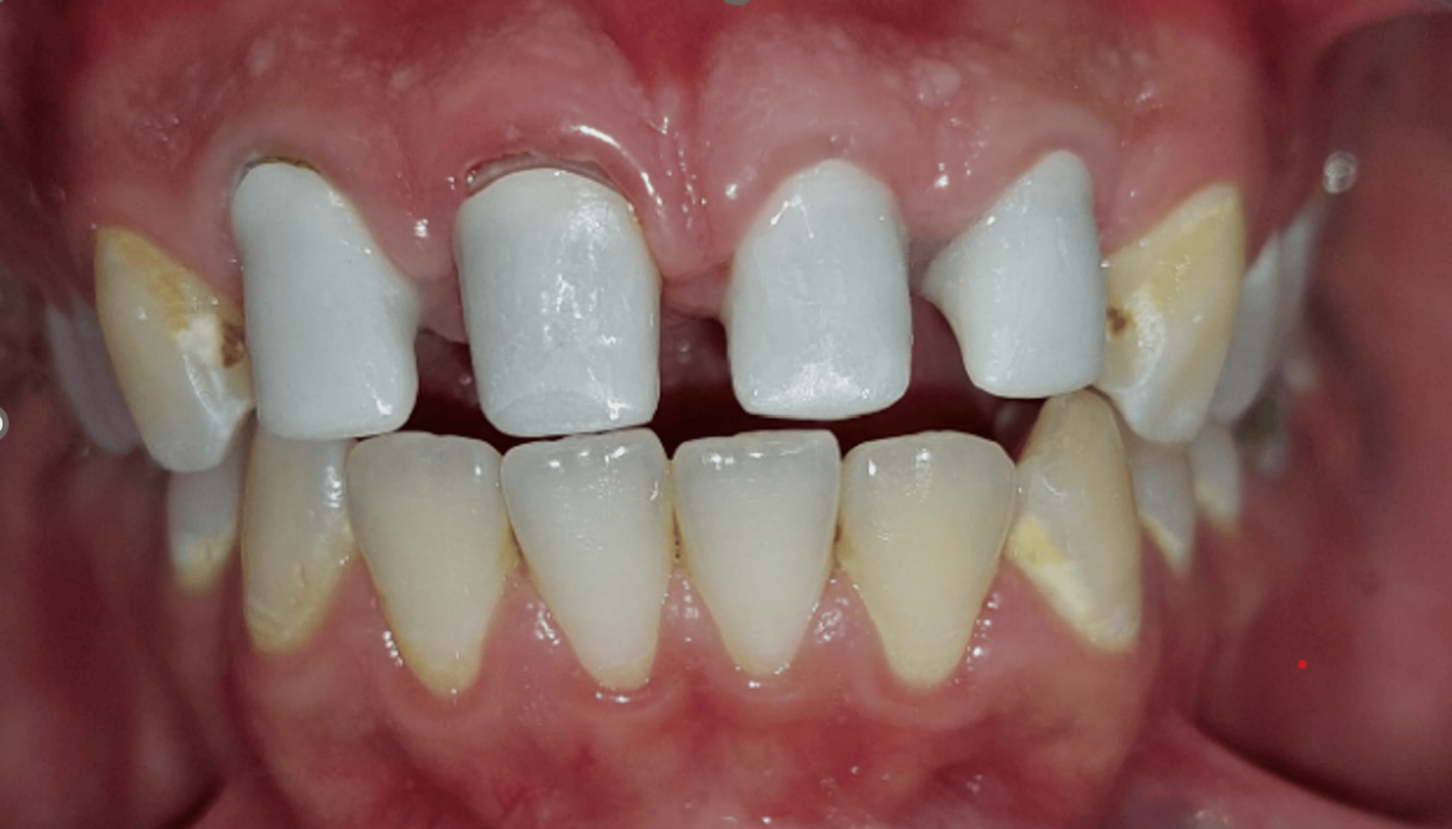
Try-in of zirconia infrastructures. Evaluation of zirconia infrastructures during the try-in phase to assess fit, marginal adaptation, and occlusion before final veneering or cementation.

After the milling of the zirconia infrastructures, a try-in was performed to assess their cervical adaptation and to ensure adequate space for the veneering ceramic. During this phase, the shade of the cosmetic ceramic was also selected to match the patient's natural dentition (Figure 12).

**Figure 13:**
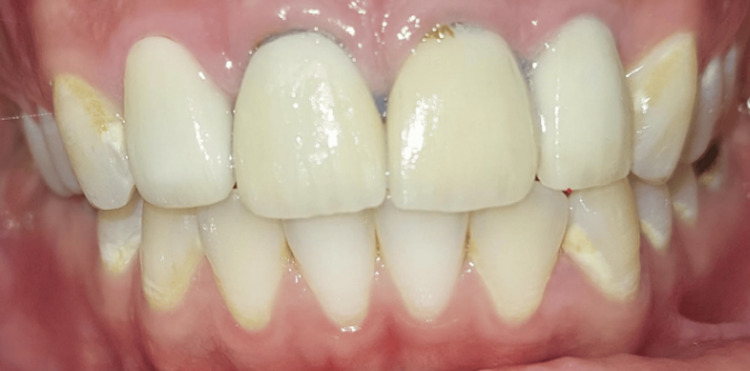
Try-in of the crowns Try-in of crowns to assess fit, occlusion, and aesthetics before final cementation.

Upon completion of the final restorations, a comprehensive evaluation was conducted to assess the occlusion, esthetics, including tooth shape, shade, and position, and the reproduction of contact points with adjacent teeth (Figure 13).

**Figure 14:**
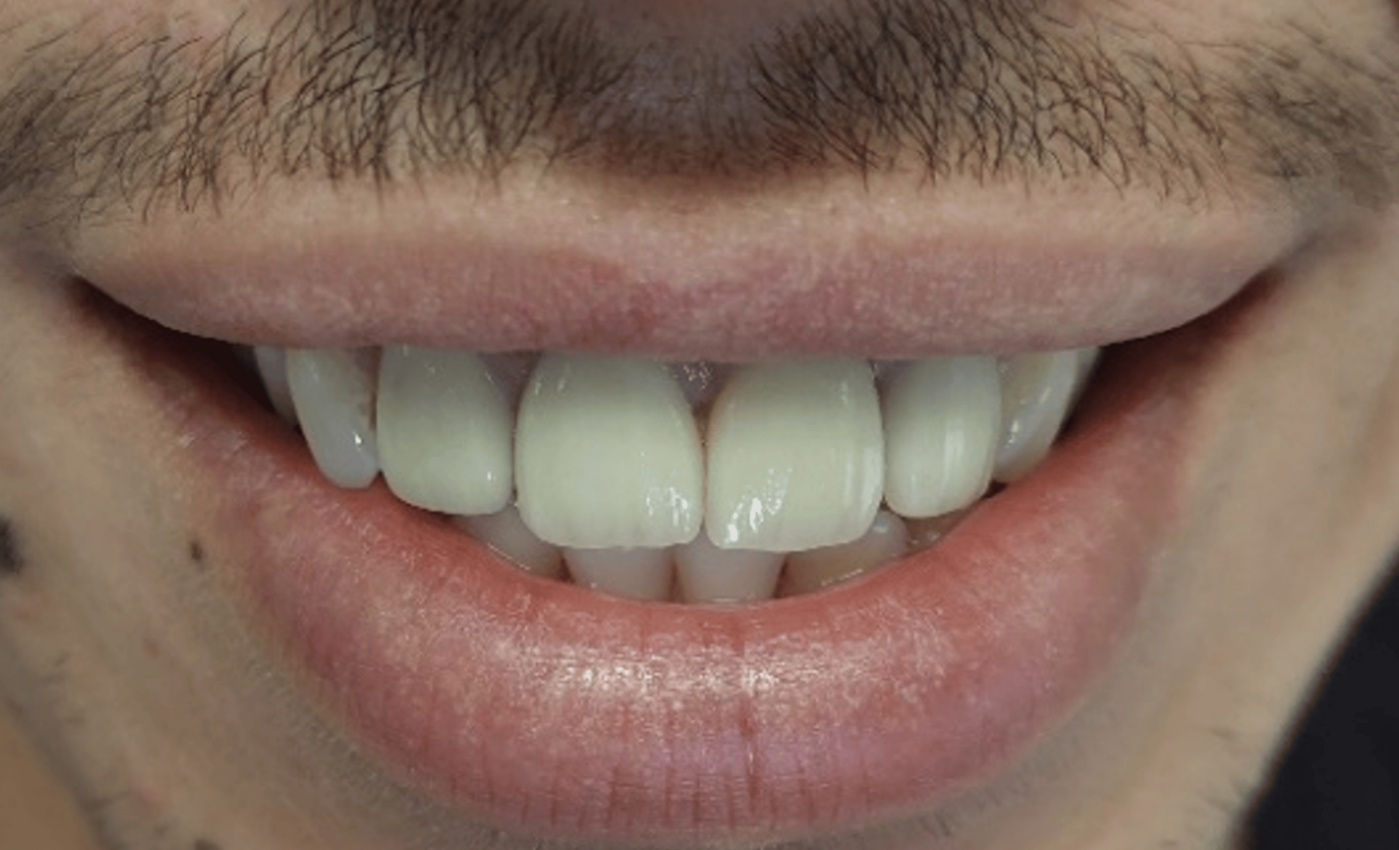
Final result. Final placement of the dental crown, showcasing optimal fit, occlusion, and aesthetics following successful try-in and adjustments.

**Figure 15:**
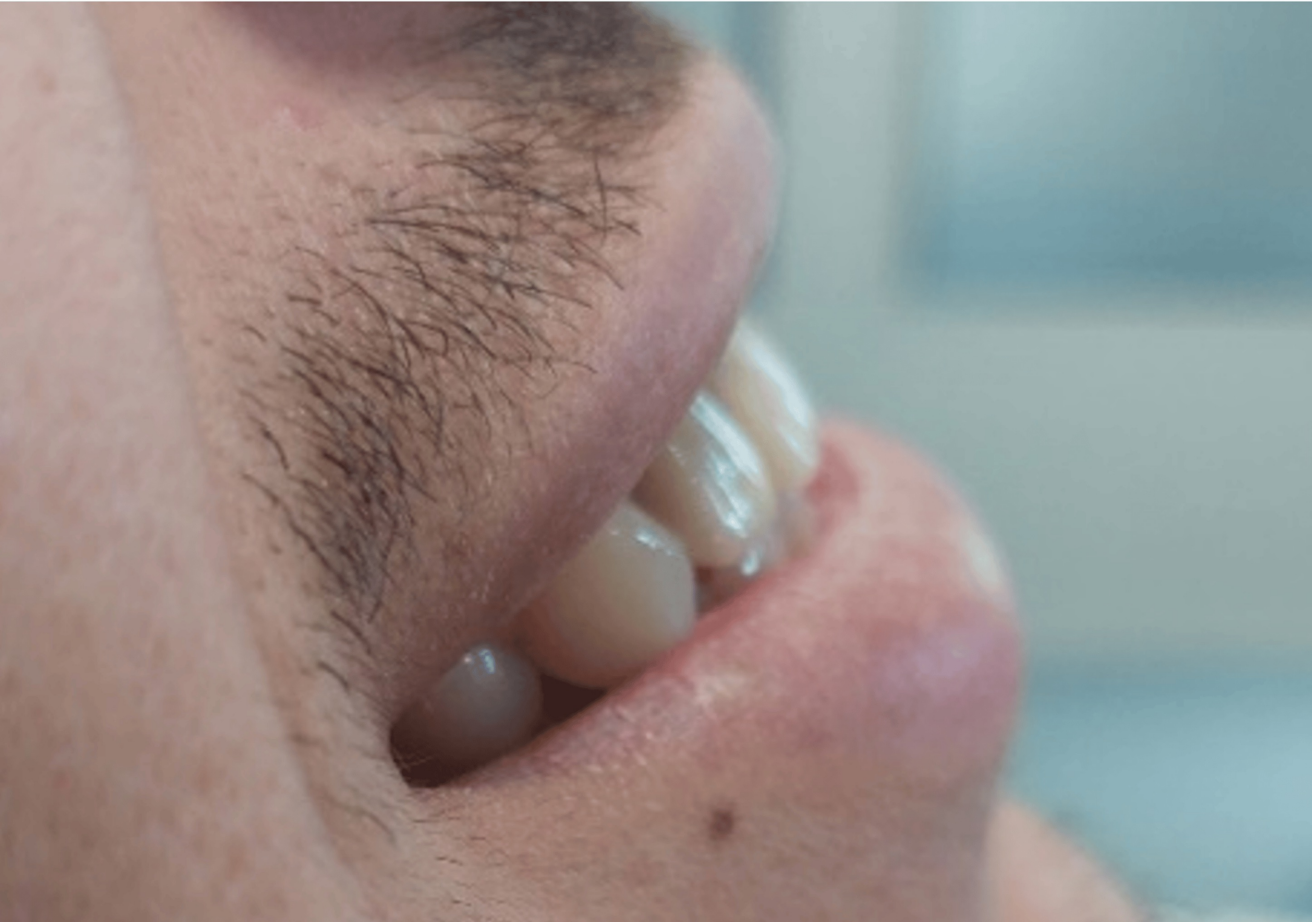
Final result in proximal view.

Cementation was performed (Figure 14) using a resin-modified glass ionomer cement to ensure a secure bond and optimal fit for the restoration (Figure 15). A follow-up appointment is scheduled six months after the placement of the final crown to assess the restoration's integration and overall performance.

## Discussion

This article illustrates the benefits of an interdisciplinary approach to the treatment of patients requiring fixed prostheses. Treatment planning must begin with visualization of the final result, before any “irreversible” aesthetic, restorative, or functional medical procedures are carried out. The “in situ” realization of the final result of an aesthetic prosthetic project is preceded by a very careful reflection phase upstream between the medical team, following a number of specific steps and responding to a precise chronology.

For this analysis, it seems necessary to use photography. Today, photography seems to be an essential element in dentistry [5]. Having a photograph means that the initial data can be preserved. But more than that, it makes it possible to reflect on the clinical situation, without the presence of the patient concerned. Analysis of each element takes time, especially when combined with additional examinations (X-rays, casts). These photographs also offer the advantage of easier communication with the laboratory technician (shade, but also axis of face, teeth, etc.). This communication allows treatment choices to be optimized, as clinical reflection is combined with technical reflection in the laboratory. However, facial analysis based exclusively on photographs is incomplete. Tarantili et al. also studied the smile on video and observed that the average duration of a spontaneous smile was 500 ms, which emphasizes the difficulty of recording this moment in photographs [6].

DSD [7] is a complementary digital tool for analyzing our patients' smiles and communicating with the laboratory. A precise protocol of photographs and associated analyses is used to outline the prosthetic project based on the initial situation. Once planning is complete, a virtual representation of the new smile can be synthesized and superimposed on the various photos to show the patient his or her new smile from different points of view.

The mock-up is the most suitable solution for validating the prosthetic project by means of a resin model transferred into the mouth using a silicone key made on the prosthetic project in question [8], providing a prefiguration of the final result. Once validated, we guide you through the various stages of treatment (dental preparations - bone and/or gum surgery).

Aesthetic crown lengthening is a surgical procedure aimed at increasing the clinical crown length and enhancing gingival contours. This intervention preserves the dentogingival complex, facilitates optimal tooth preparation, ensures a secure marginal seal for restorative procedures (=joint marginal), and improves mechanical retention for the final crowns [8]. This treatment usually requires diagnostic information, such as a periodontal record, radiographic evaluation, diagnostic wax-ups, and a mock-up. The role of symmetrical gingival tissue with a correctly positioned zenith is essential in achieving esthetic prosthetic treatment. 

Studies have demonstrated that placing restorative margins below the gingiva can lead to early gingival recession and loss of attachment, particularly in areas where the width of attached gingiva is less than 3 mm [9]. This underscores the importance of considering gingival health when planning restorative procedures.

Research indicates that intrasulcular margins, especially in the presence of minimal or no attached gingiva, can negatively impact gingival health. These conditions may lead to chronic inflammation and increased susceptibility to further recession. Therefore, gingival augmentation is recommended in sites where intrasulcular restorative margins are anticipated, notably in anterior areas, to preserve periodontal health and esthetics.

In clinical practice, it is essential to evaluate the width of attached gingiva before proceeding with restorative treatments. If the attached gingiva is insufficient, procedures such as gingival augmentation may be indicated to enhance the zone of keratinized tissue [10], thereby reducing the risk of recession and ensuring the long-term success of restorative interventions.

CAD/CAM [11] technology has transformed modern dentistry by enhancing precision, efficiency, and patient outcomes. This integrated system encompasses digital scanning, advanced software, and automated milling to streamline the creation of dental restorations (CAD, CAM), enabling analog clinical data to be recorded (acquisition) in digital form, and virtual modeling (CAD) and material production (CAM) of a customized medical device.

CAD/CAM has made it possible to improve several aspects of prosthesis quality, in particular by reducing the risk of error through the abandonment of traditional impression techniques for direct and semi-direct CAD/CAM and the elimination of numerous empirical steps. This improves the precision of prostheses [12]. It also offers a large quantity of archival and unalterable data, with no storage time and no risk of infectious contamination. As opposed to plaster models.

Aesthetics are also improved by the interaction of several parameters, including the precision of digital tools [13], the planning and previewing of the aesthetic project by both practitioner and patient, materials with improved optical properties, and better communication between practitioner and laboratory.

The digitization of dental laboratories has revolutionized the habits of dental technicians, while giving them access to new, innovative, and varied materials with remarkable mechanical and aesthetic properties to satisfy all types of restoration. By reducing human time and manual handling, the use of digital processes considerably increases productivity.

Digitized means of communication open up new possibilities for laboratories and practices to exchange information even more efficiently and in real time.

Digital imaging such as digital volumetric tomography and 3D facial scanning enables dentists to provide their dental technicians with a rich wealth of patient data. This enables them to achieve high-quality results [13].

## Conclusions

This clinical report focused on the multidisciplinary approach required to manage anterior aesthetic projects. In-depth knowledge of the relationship between periodontal tissue and restorative dentistry is essential to the guarantee of form, function, aesthetics and health of dental tissues, while the involvement of digital tools has enabled the realization of prosthetic reconstructions with high degrees of precision.

## References

[1] Muts EJ, van Pelt H, Edelhoff D, Krejci I, Cune M (2014). Tooth wear: a systematic review of treatment options. J Prosthet Dent.

[2] Jafri Z, Ahmad N, Sawai M, Sultan N, Bhardwaj A (2020). Digital Smile Design-an innovative tool in aesthetic dentistry. J Oral Biol Craniofac Res.

[3] de Matos JD, Nakano LJ, Lopes G (2021). Post and core: a new clinical perspective - myths and facts. Arch Health Invest.

[4] Abduo J, Palamara JE (2021). Accuracy of digital impressions versus conventional impressions for 2 implants: an in vitro study evaluating the effect of implant angulation. Int J Implant Dent.

[5] Stanley M, Paz AG, Miguel I, Coachman C (2018). Fully digital workflow, integrating dental scan, smile design and CAD-CAM: case report. BMC Oral Health.

[6] Tarantili VV, Halazonetis DJ, Spyropoulos MN (2005). The spontaneous smile in dynamic motion. Am J Orthod Dentofacial Orthop.

[7] Lee EA, Cambra V, Bergler M (2024). Staged esthetic crown lengthening: classification and guidelines for periodontal-restorative therapy. J Esthet Restor Dent.

[8] Jurado CA, Parachuru V, Villalobos Tinoco J, Guzman-Perez G, Tsujimoto A, Javvadi R, Afrashtehfar KI (2022). Diagnostic mock-up as a surgical reduction guide for crown lengthening: technique description and case report. Medicina (Kaunas).

[9] Qali M, Alsaegh H, Alsaraf S (2024). Clinical considerations for crown lengthening: a comprehensive review. Cureus.

[10] Ganji KK, Patil VA, John J (2012). A comparative evaluation for biologic width following surgical crown lengthening using gingivectomy and ostectomy procedures. Int J Dent.

[11] Miyazaki T, Hotta Y, Kunii J, Kuriyama S, Tamaki Y (2009). A review of dental CAD/CAM: current status and future perspectives from 20 years of experience. Dent Mater J.

[12] Suganna M, Kausher H, Tarek Ahmed S (2022). Contemporary evidence of CAD-CAM in dentistry: a systematic review. Cureus.

[13] Padrós R, Giner L, Herrero-Climent M, Falcao-Costa C, Ríos-Santos JV, Gil FJ (2020). Influence of the CAD-CAM systems on the marginal accuracy and mechanical properties of dental restorations. Int J Environ Res Public Health.

